# A minimalist fluorescent probe for differentiating Cys, Hcy and GSH in live cells†
†Electronic supplementary information (ESI) available: Synthesis, spectroscopic properties, NMR, mass spectra and confocal imaging. CCDC 1402255. For ESI and crystallographic data in CIF or other electronic format see DOI: 10.1039/c5sc02431e



**DOI:** 10.1039/c5sc02431e

**Published:** 2015-09-22

**Authors:** Huatang Zhang, Ruochuan Liu, Jie Liu, Lin Li, Ping Wang, Shao Q. Yao, Zhengtao Xu, Hongyan Sun

**Affiliations:** a Department of Biology and Chemistry , City University of Hong Kong , 83 Tat Chee Avenue , Kowloon , Hong Kong , China . Email: hongysun@cityu.edu.hk ; Email: zhengtao@cityu.edu.hk; b Key Laboratory of Biochip Technology, Biotech and Health Centre , Shenzhen Research Institute of City University of Hong Kong , Shenzhen , 518057 , PR China; c Key Laboratory of Flexible Electronics & Institute of Advanced Materials , Nanjing Tech University , 30 South Puzhu Road , Nanjing , 211816 , China; d Department of Chemistry , National University of Singapore , 117543 , Singapore

## Abstract

A simple molecule, tetrafluoroterephthalonitrile (4F-2CN), was discovered to be an efficient fluorescent probe for detecting biological thiol species. The probe responded to Cys and emitted strong green fluorescence, whereas it reacted with Hcy/GSH and generated blue fluorescence. Addition of CTAB (cetyl trimethylammonium bromide) was observed to alter the fluorescence color of the reaction product of 4F-2CN and Hcy (from blue to green), but no alteration of the fluorescence color occurred for Cys and GSH. For the very first time, cell imaging experiments showed that the three commonly occurring thiols (Cys/Hcy/GSH) could be differentiated using a single fluorescent probe. In addition, the reaction product of 4F-2CN and Cys exhibits two-photon properties, offering a potentially useful tool for tissue imaging studies. To the best of our knowledge, 4F-2CN is currently the smallest fluorescent probe for thiol detection. We envision that this new and versatile probe will be a useful tool for further elucidating the roles of thiols in biology.

## Abstract 2

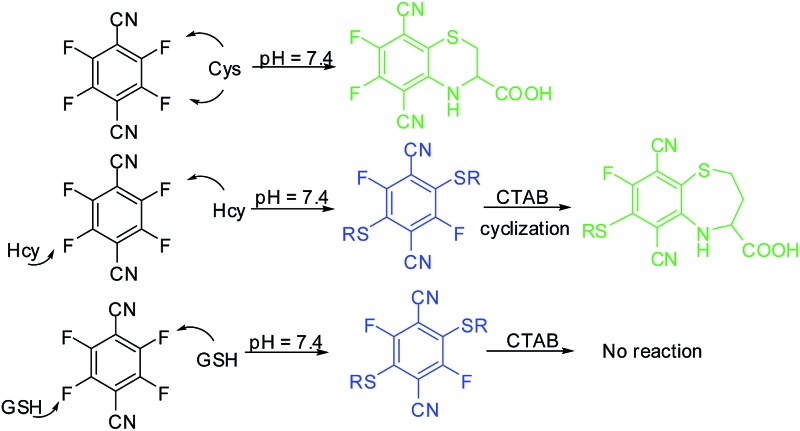
A simple and versatile probe was discovered for discriminating between different thiol species with the use of CTAB (cetyl trimethylammonium bromide).

## Introduction

Small molecule thiols play crucial roles in maintaining cellular redox environments and mitigating damage from free radicals and toxins.^1^ Three low-molecular-weight thiols are commonly found in biological systems, namely cysteine (Cys), homocysteine (Hcy) and glutathione (GSH). These thiols are closely involved in regulating various physiological and pathological processes. For example, Cys is an essential amino acid for protein synthesis. Abnormal levels of Cys are related to slow growth, edema, lethargy, liver damage, *etc.* ^2^ Hcy elevation in plasma, on the other hand, is a risk factor for cardiovascular disease, Alzheimer's disease and osteoporosis,^3^ while GSH is closely linked to leucocyte loss, cancer, HIV infection, *etc.* ^4^ The important biological roles of thiols have thus spurred strong interest in developing useful chemical tools for detecting thiols.

The use of fluorescent probes, owing to their simplicity and non-invasiveness, has become a popular approach for thiol detection in living cells.^5^ Over the last decade, a large number of fluorescent probes have been developed for detecting thiols.^6^ Most of these probes are based on thiol-selective chemical reactions, including Michael addition reactions,^7^ nucleophilic substitution,^8^ cyclization reactions between aldehyde and aminothiols,^9^ cleavage reactions of 2,4-dinitrobenzenesulfonyl (DNBS) with thiols,^10^ disulfide exchange reaction,^11^ and others.^12^

**Fig. 1:**
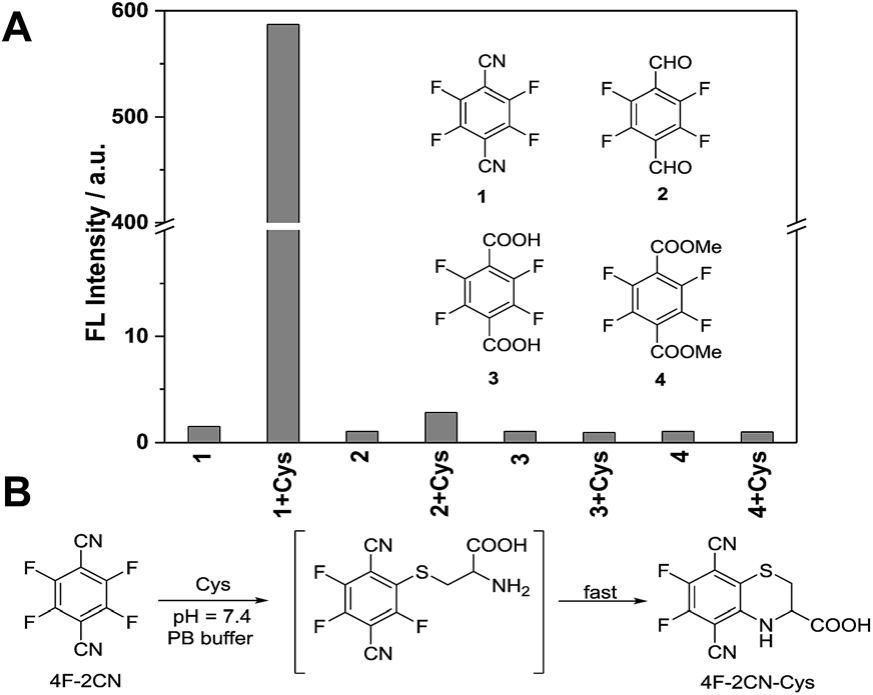
(A) Fluorescence response of 4F-2CN and its analogues after incubation with Cys in PB buffer (Ex/Em at 420 nm/500 nm). (B) Proposed reaction mechanism of 4F-2CN and Cys.

Similarities among the structures and the reaction activities of Cys, Hcy and GSH have posed substantial difficulties for discriminating one thiol species from another. Despite this challenge, several fluorescent probes that allow for the selective detection of Cys or GSH have been reported, *e.g.*, the Strongin group's seminal work on the selective detection of Cys/Hcy over GSH using a cyclization reaction between Cys/Hcy and acrylate.^13^ Yang and coworkers also designed a GSH-selective probe by employing the specific thiol–halogen reaction between chlorinated BODIPY and GSH.^14^ Further effort has been devoted recently to develop single probe systems for simultaneous detection of two or three thiol species.^15^ For example, Guo and coworkers have developed a chlorinated coumarin–hemicyanine probe for the simultaneous detection of Cys and GSH. Nevertheless, single probes that are able to fully discriminate the three thiols from each other are still quite rare. In this study, we report a remarkably simple but versatile probe 4F-2CN (Fig. 1), which is capable of simultaneously detecting Cys and Hcy/GSH using dual emission channels. Moreover, the fluorescence color for Hcy and the probe can be altered by adding a surfactant called CTAB, thereby allowing all three thiols to be differentiated from each other.

## Results and discussion

In a screening experiment, we identified a commercial compound, 2,3,5,6-tetrafluoroterephthalonitrile (4F-2CN), which was shown to react with Cys and produce bright green fluorescence in PB buffer (10 mM phosphate buffer, pH = 7.4) (Fig. 1A). It was noted that when the CN groups were altered to other electron withdrawing groups like CHO/COOMe, no obvious fluorescence was observed with these analogues. TLC experiments also showed that no reaction occurred between the analogues and Cys under PBS/DMF = 1 : 1 after 2 h (Fig. S1†). The possible reason for the different reactivity of these compounds could be due to the difference in the electron-withdrawing abilities of CN, CHO and COOMe. With non-thiol amino acids (*e.g.*, histidine and lysine), no reaction with 4F-2CN occurred as indicated by fluorescence assays and mass spectrometry analysis.

We characterized the reaction product of 4F-2CN and Cys. 4F-2CN was mixed with Cys in a DMF solution to give the product 4F-2CN–Cys which was characterized using ^1^H, ^13^C and ^19^F NMR, ESI-MS, FT-IR and X-ray crystallography of single crystals (CCDC ; 1402255; Fig. S2–S8, Tables S1–S3†). We hypothesize that the formation of 4F-2CN–Cys was initiated by the Cys thiol group replacing a fluoro group on 4F-2CN in an aromatic nucleophilic substitution, and the subsequent cyclization was facilitated by the six-membered ring configuration afforded by the Cys substrate (Fig. 1B). The strong fluorescence from the cyclized product can be ascribed largely to the electron donation from the amino and sulfide moieties onto the aromatic core. We also determined the quantum yield of 4F-2CN–Cys to be 0.35 (Table S4†), indicating that it is a good fluorophore.

**Fig. 2:**
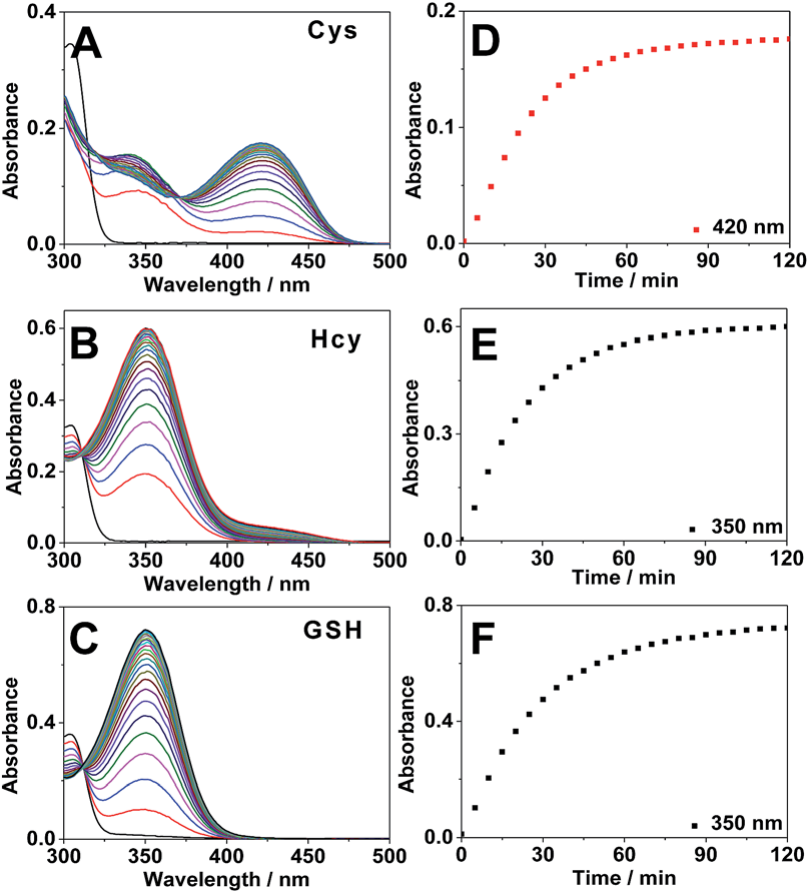
(A–C) Time-dependent absorption spectra of 4F-2CN (50 μM) incubated with 1 mM of Cys (A), Hcy (B) and GSH (C) in PB buffer (10 mM, pH 7.4). (D–F) Corresponding absorbance changes of 4F-2CN incubated with Cys (D), Hcy (E) and GSH (F).

Next we carried out time-dependent absorbance experiments with the probe. 4F-2CN alone in PB buffer did not show any absorption in the range of 340–500 nm (Fig. S9†). Upon addition of Cys, a new absorption peak at 420 nm was immediately observed, and the absorption signal reached equilibrium after around 2 h (Fig. 2A and D). For Hcy and GSH, an absorption peak at 350 nm could be observed, and it also plateaued after around 2 h (Fig. 2B, C, E and F).

**Fig. 3:**
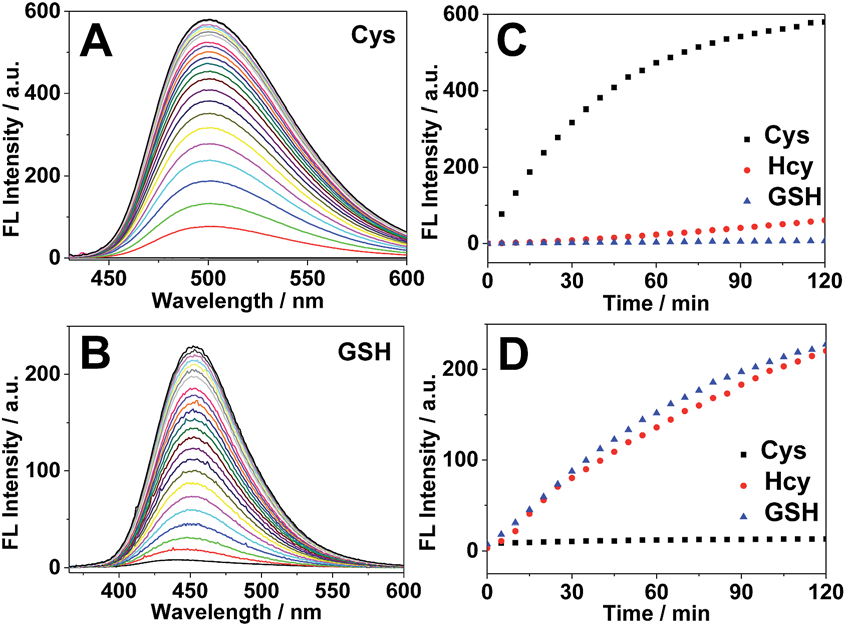
(A and B) Time-dependent fluorescence spectra of 4F-2CN (10 μM) with 100 μM of Cys (A), GSH (B) excited at 350 nm and 420 nm respectively. (C and D) Corresponding fluorescence intensity changes of 4F-2CN with Cys/Hcy/GSH excited at 350 nm and 420 nm respectively.

Subsequently we carried out the time-dependent fluorescence experiments of 4F-2CN with Cys, Hcy and GSH respectively. The fluorescence signal was first measured at an excitation wavelength of 420 nm. The probe itself showed negligible fluorescence. Upon addition of Cys, a new emission peak at 500 nm was readily observed and reached equilibrium in around 2 h (Fig. 3A). In comparison with Cys, Hcy displayed only a slight fluorescence increment and GSH did not show a noticeable signal (Fig. 3C). The fluorescence increments of 4F-2CN at 500 nm for Cys, Hcy and GSH are 400-, 30- and 4.5-fold respectively. The results indicated that 4F-2CN could be used to discriminate Cys from Hcy/GSH when excited at 420 nm. Furthermore, we performed titration experiments of 4F-2CN with the addition of different concentrations of Cys. The results indicated that the fluorescence intensity was nearly linear to the Cys concentration in the range of 0–20 μM (Fig. S12†). The detection limit of the probe for Cys was determined to be as low as 20 nM based on the 3*σ*/slope method. Moreover, an 8.7-fold fluorescence increase was observed when the Cys concentration was 1 μM. These results demonstrated that 4F-2CN could serve as a highly sensitive probe for detecting Cys.

The fluorescence response of 4F-2CN with Cys/Hcy/GSH was then investigated using an excitation wavelength of 350 nm. Similar to the previous study, the probe itself displayed negligible fluorescence in this channel. Upon addition of GSH or Hcy, a new fluorescence emission peak at 450 nm appeared (Fig. 3B and S10†). On the other hand, Cys did not show an obvious fluorescence increment at 450 nm (Fig. 3D). The fluorescence increments at 450 nm for Hcy, GSH and Cys are 29-, 26- and 2.7-fold respectively. The results demonstrated that the probe was able to differentiate GSH/Hcy from Cys when excited at 350 nm. Concentration-dependent experiments with the probe indicated that the fluorescence signal at 450 nm increased with increased concentrations of Hcy or GSH (Fig. S13 and S14†). The detection limits of Hcy and GSH were determined to be 2.27 and 0.24 μM respectively. It is noticed that the LOD of Cys was about ten times lower than that of Hcy/GSH. The higher sensitivity observed for Cys arises from the strong fluorescence of the reaction product of Cys and 4F-2CN. The enhanced fluorescence in the case of Cys can be attributed to the electron-donating amino group, as well as the rigid cyclized structure in the product. In addition, the reaction with Cys proceeded faster than that with Hcy/GSH (Fig. 3C and D), contributing to the higher sensitivity for Cys observed within the given length of time.

**Fig. 4:**
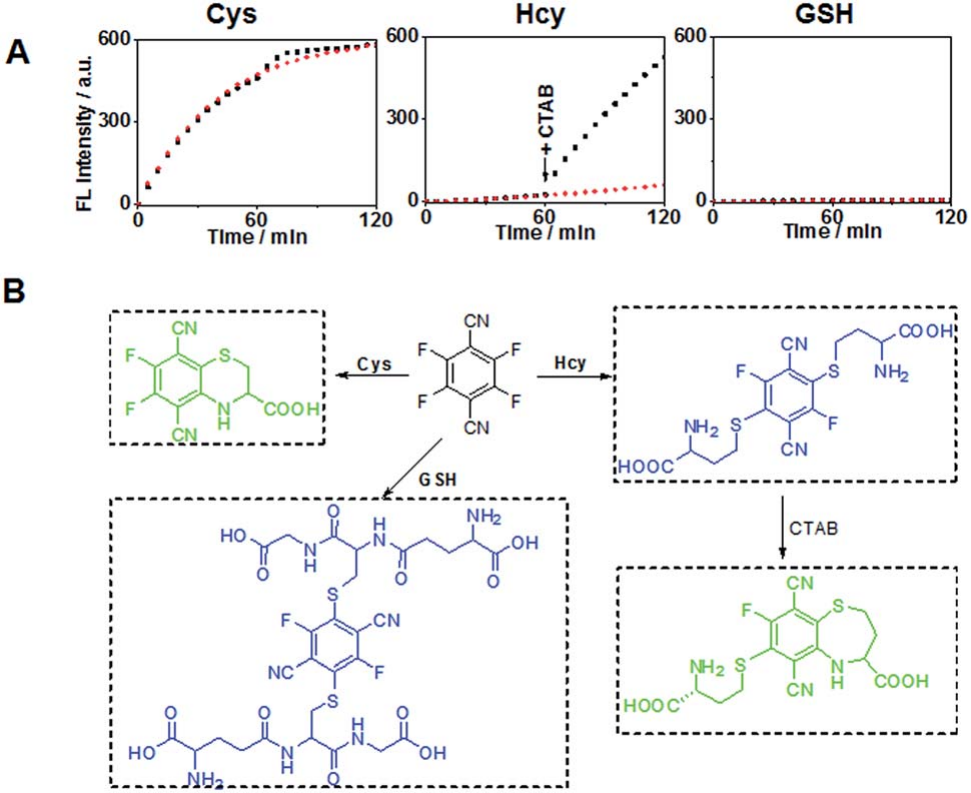
(A) Fluorescence intensity changes of 4F-2CN reacting with Cys, Hcy and GSH with/without addition of CTAB buffer (Ex/Em at 420 nm/500 nm). The red lines represent the reaction without the addition of CTAB. The black lines denote the event that CTAB was added to the reaction after 1 h of incubation. (B) Schemes for 4F-2CN reacting with Cys, Hcy and GSH respectively.

Previous studies have shown that the surfactant CTAB can help to form micelles and facilitate the intramolecular ring formation.^16^ In our study, it was found that the fluorescence properties of the reaction between 4F-2CN and Hcy could be altered by adding CTAB. As shown in Fig. 4A, a weak fluorescence signal was observed at 500 nm when 4F-2CN was incubated with Hcy for 1 h. After CTAB was added, a gradual fluorescence increase could be readily observed at 500 nm. On the other hand, the reactivity of 4F-2CN with Cys and GSH remained almost unchanged after the addition of CTAB. From these data, we can draw the conclusion that 4F-2CN displayed distinct reactivity patterns towards Cys, Hcy and GSH by using CTAB. The probe can be potentially used to differentiate these three highly similar thiol species. We further characterized the reaction products of 4F-2CN with Hcy and GSH using HPLC, ESI-MS and NMR. Experimental data revealed that disubstituted products were produced in the reaction of 4F-2CN and Hcy/GSH (Fig. 4B and S15–S24†). Addition of CTAB will facilitate the cyclization of 4F-2CN and Hcy, but not the cyclization of 4F-2CN and GSH (Fig. S18†).

**Fig. 5:**
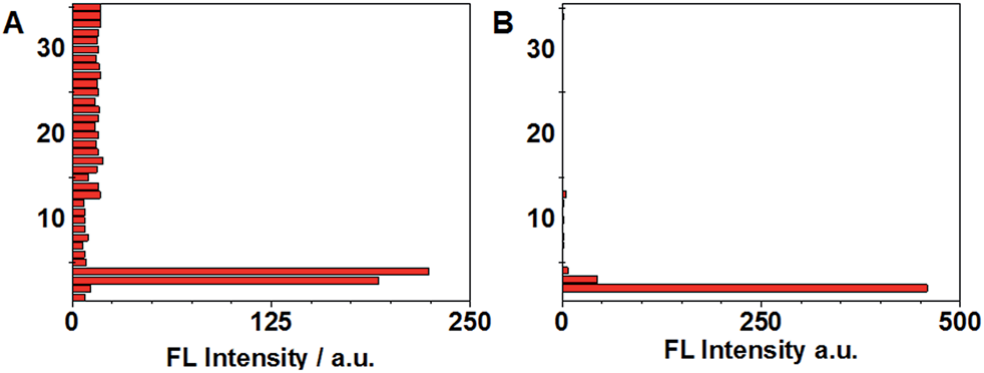
(A and B) Selectivity studies of 4F-2CN with various biological analytes at two different emission wavelengths. (A) *λ*_ex_ = 350 nm, *λ*_em_ = 450 nm; (B) *λ*_ex_ = 420 nm, *λ*_em_ = 500 nm. (1) control; (2) Cys; (3) Hcy; (4) GSH; (5) K^+^; (6) Na^+^; (7) Mg^2+^; (8) Zn^2+^; (9) NO_2_^–^; (10) N_3_^–^; (11) S_2_O_3_^2–^; (12) S^2–^; (13) ˙OH; (14) ˙OtBu; (15) H_2_O_2_; (16) TBHP; (17) Ala; (18) Ile; (19) Leu; (20) Val; (21) Phe; (22) Try; (23) Tyr; (24) Asn; (25) Gln; (26) Met; (27) Ser; (28) Thr; (29) Asp; (30) Glu; (31) Arg; (32) His; (33) Lys; (34) Gly; (35) Pro.

Selectivity experiments are instrumental to ascertain the biological applications of the probe, such as cell imaging experiments. The selectivity experiments were performed by incubating the probe with various biological analytes, including 20 natural amino acids, Hcy, GSH, H_2_S, RNS, ROS and metal ions. The fluorescence intensities were then measured at two different emission wavelengths. As shown in Fig. 5A, the fluorescence intensity at 450 nm showed that only GSH/Hcy induced significant fluorescence changes whereas the other biological analytes gave marginal increments. On the other hand, the fluorescence intensity for Cys at 500 nm displayed a substantial increment; Hcy showed small increment and the other samples induced very little increment (Fig. 5B). These results indicated that the probe is highly selective toward thiols.

**Fig. 6:**
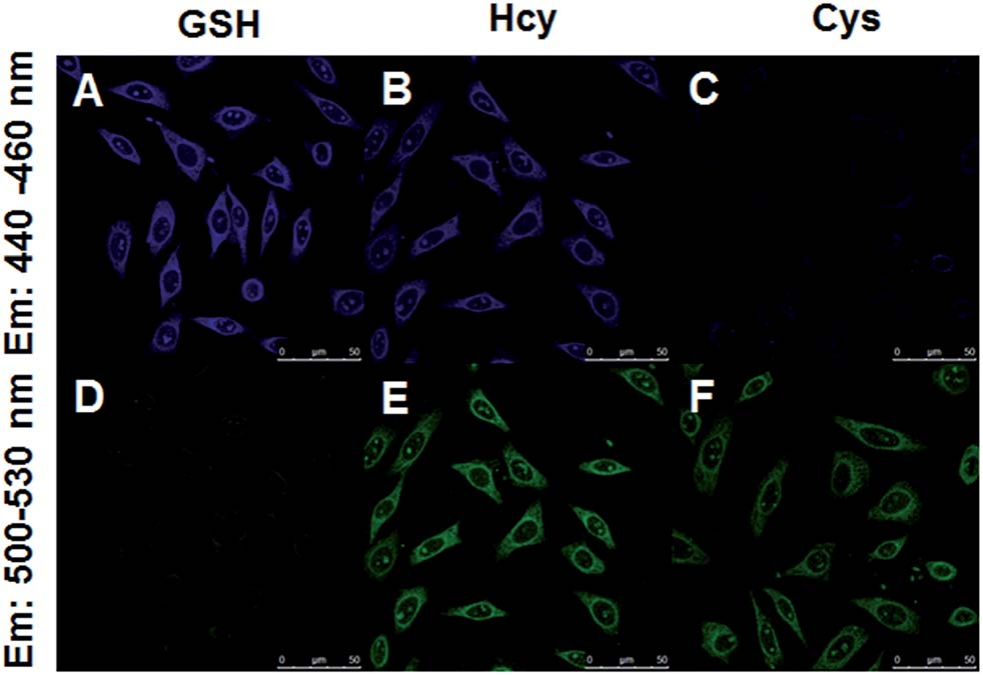
Fluorescence microscopy experiments for imaging Cys/Hcy/GSH with 4F-2CN using dual emission channels (green channel: emission was collected at 500–530 nm, blue channel: emission was collected at 440–460 nm. Both channels were excited at 405 nm). HeLa cells were first incubated with CTAB for 5 min, then NEM (2 mM) for 20 min. Subsequently Cys/Hcy/GSH (5 mM) was added and incubated for 15 min. 4F-2CN was then added and incubated for another 20 min. (A) Cells with NEM and GSH (blue channel), (B) cells with NEM and Hcy (blue channel), (C) cells with NEM and Cys (blue channel), (D) cells with NEM and GSH (green channel), (E) cells with NEM and Hcy (green channel), (F) cells with NEM and Cys (green channel).

Encouraged by the above results, we moved forward to study the capability of 4F-2CN to image Cys/Hcy/GSH in living cells. HeLa cells were firstly treated with CTAB. *N*-Ethylmaleimide (NEM), a common thiol depletion reagent was then added. Cys, Hcy and GSH were subsequently added to the medium respectively. 4F-2CN was finally added to the medium and incubated for 20 min. Confocal imaging results showed that cells with the addition of Cys displayed green fluorescence (Fig. 6C and F). GSH treated cells, on the other hand, gave off bright blue fluorescence (Fig. 6A and D). Cells treated with Hcy showed both blue and green fluorescence (Fig. 6B and E). The green fluorescence is attributed to the cyclized product of the reaction between 4F-CN and Hcy. These results together unambiguously proved that 4F-2CN could be used to differentiate between Cys, Hcy and GSH *via* dual emission channels. We also performed MTT assays to examine the cytotoxicity of 4F-2CN. As shown in Fig S26,† the probe showed relatively low toxicity at 10 μM (this is the concentration we used for the cell imaging studies in this work). Higher toxicity was observed with higher concentrations. Thus it is recommended to keep the probe concentration equal to or below 10 μM.

**Fig. 7:**
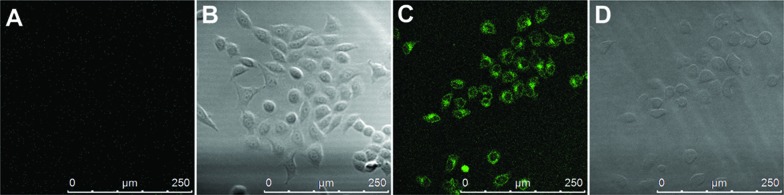
Two-photon fluorescence imaging studies. (A) Two-photon image of cells only; (B) bright field image of cells only; (C) two-photon image of cells with the addition of 4F-2CN; (D) bright field image of cells with the addition of 4F-2CN.

In addition, we found that 4F-2CN–Cys possesses two-photon properties when excited at 860 nm (Fig. S27†), whereas no two-photon signal from the reaction product of 4F-2CN and Hcy/GSH was observed. Further cell imaging experiments showed that living cells displayed fluorescence under two-photon excitation after 4F-2CN was added (Fig. 7). This study indicated that 4F-2CN might serve as a useful two-photon probe for selective detection of Cys in tissue imaging studies. The reason that 4F-2CN–Cys has two-photon properties could be attributed to the following two factors. First, 4F-2CN–Cys has strong electron withdrawing (CN) and electron donating (NH) groups. In general, adding strong electron donors and acceptors to the conjugated π system can enhance two-photon signals.^17^ For 4F-2CN–Hcy and 4F-2CN–GSH, the electron donating ability of the thiol group is less effective compared with that of the amine moiety. Second, it was observed that a rigid conformation can also enhance two-photon properties.^17^ 4F-2CN–Cys forms a cyclized product, which increases the rigidity of the product's conformation, whereas 4F-2CN–Hcy and 4F-2CN–GSH do not.

Finally, we examined the fluorescence response of 4F-2CN with three sets of thiol mixtures, Cys/Hcy, Cys/GSH and Hcy/GSH. As shown in Fig. S28,† the detection of Cys is highly sensitive. It can be selectively detected even in the presence of 10 eq. of Hcy or GSH (*λ*_ex_ = 420 nm). On the other hand, in the Cys/Hcy and Cys/GSH mixtures, Hcy (as well as GSH) can also be detected without much interference from Cys in the mixture (*λ*_ex_ = 350 nm). As for the Hcy/GSH mixture, the two thiols cannot be differentiated when excited at 350 nm. However, selective detection of Hcy can be achieved through the addition of CTAB, and the Hcy signal was not interfered with in the presence of GSH.

## Conclusion

In this study, we have discovered a novel and remarkably simple probe, 4F-2CN, which could undergo selective reactions with thiols under physiological conditions. Importantly, 4F-2CN displayed distinct reaction profiles for Cys, Hcy and GSH with the use of CTAB. Our bioimaging experiments proved, for the very first time, that Cys, Hcy and GSH could be differentiated using a single fluorescent probe. It should be noted that the probe can be subjected to further chemical modification and produce derivatives with different photophysical properties. The research work for synthesizing different derivatives of 4F-2CN is currently in progress. Interestingly 4F-2CN–Cys was found to possess two-photon properties, whereas the reaction product of 4F-2CN and Hcy/GSH does not possess any two-photon properties. Cell imaging experiments showed that two-photon fluorescence could be observed with the addition of 4F-2CN. The results indicated that the probe could serve as a useful tool for the selective detection of Cys in tissue imaging studies. Given the small size and excellent properties of 4F-2CN, we envision that this new and versatile probe will be a useful tool for further elucidating the roles of thiols in biology.
