# Supplementary material for: A minimalist fluorescent probe for differentiating Cys, Hcy and GSH in live cells
Source: Chem Sci. 2015 Sep 22;7(1):256–60. doi: 10.1039/c5sc02431e (PMC5515046; doi:10.1039/c5sc02431e)
Supplement: Supplementary file 1 [file SC-007-C5SC02431E-s001.pdf]

Electronic Supplementary Material (ESI) for Chemical Science.
This journal is © The Royal Society of Chemistry 2015

Supplementary Information

A Minimalist Fluorescent Probe for Differentiating Cys, Hcy and GSH in Live Cells

Huatang Zhang[a,b], Ruochuan Liu[a], Jie Liu[a], Lin Li[c], Ping Wang[a], Shao Q. Yao[d], Zhengtao Xu[a*] & Hongyan Sun[a,b*]

[a] Department of Biology and Chemistry, City University of Hong Kong, 83 Tat Chee Avenue, Kowloon, Hong Kong, China.

[b] Key Laboratory of Biochip Technology, Biotech and Health Centre, Shenzhen Research Institute of City University of Hong Kong, Shenzhen, P. R. China, 518057.

[c] Key Laboratory of Flexible Electronics & Institute of Advanced Materials, Nanjing Tech University, 30 South Puzhu Road, Nanjing, 211816, China.

[d] Department of Chemistry, National University of Singapore, 117543, Singapore.

Corresponding author: Hongyan Sun & Zhengtao Xu

Email: hongysun@cityu.edu.hk & zhengtao@cityu.edu.hk

1. Material and Methods

1.1 Material and instruments

All chemicals and solvents were purchased from commercial suppliers with the highest grade. Dulbecco's modified Eagle's medium (DMEM), fetal bovine serum (FBS), trypsin-EDTA and penicillin/streptomycin were purchased from Invitrogen. Chemicals were used directly in the experiment without further purification, unless otherwise specified. All reactions were monitored by TLC on pre-coated silica plates (Merck 60 F254, 250 μm thickness), and spots were visualized by UV light or iodine. Merck silica gel 60 (70-200 mesh) was used for column chromatography purification. ^{1}H NMR and ^{13}C NMR spectra were recorded on a Bruker NMR spectrometer (400 MHz or 300 MHz). Chemical shifts are reported in parts per million relative to internal standard tetramethylsilane ($Si(CH_3)_4$ = 0.00 ppm) or residual solvent peaks (DMSO-d_6 = 2.50 ppm, CD_3OD = 3.31 ppm). ^{1}H NMR coupling constants (*J*) are reported in Hertz (Hz), and multiplicity is indicated as follows: s (singlet), d (doublet), t (triplet), m (multiplet), dd (doublet of doublet). Mass spectra were obtained on a PC Sciex API 150 EX ESI-MS system using electrospray ionization (ESI). Infrared (IR) spectra were recorded on a Perkin Elmer Spectrum 1000. For liquids and oils samples, thin films on NaCl plates were used. For solids and crystals, KBr discs were used. Crystal data were collected on an Oxford Diffraction Gemini S Ultra X-ray single-crystal diffractometer. The structures were solved using direct methods and refined by the SHELXL-97 program. UV absorption spectra were obtained on Shimadzu 1700 UV/Vis Spectrometer. Fluorescence signal was recorded with a FluoroMax-4 fluorescence photometer. Fluorescence images were acquired using a Leica TCS SP5 or SPE Confocal Scanning Microscope. pH value was recorded with a FiveEasy ™

Fe20 pH meter. HPLC analysis was performed on a Waters column (4.6 mm × 250 mm, Symmetryshield™ RP18) using a Waters 2489 HPLC system. Flow rate was set at 1.0 mL/min.

1.2 Synthesis of 4F-2CN-Cys

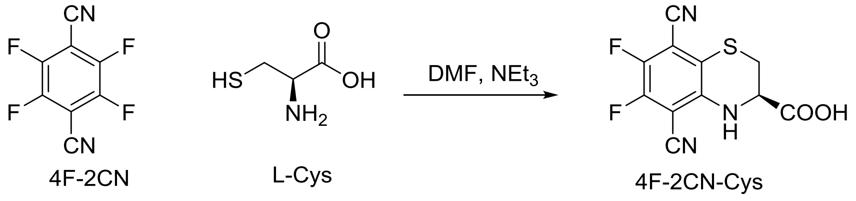

4F-2CN (200 mg, 1.0 mmol) and L-Cysteine (605 mg, 5.0 mmol) were first dissolved in 10 mL of DMF. Triethylamine (278 μL, 2 mmol) was then added to the DMF solution. The mixture was stirred at room temperature for 30 min and the reaction solution changed to yellow color. Water was added to the reaction mixture and a white precipitate appeared. The pH value of the solution was adjusted to 6 with 6M HCl. The resulting solution was then filtered to obtain a yellow solid (241 mg) with 86% yield. ^{1}H NMR (300 MHz, DMSO-d_6) δ 13.29 (s, 1H), 7.63 (d, J = 3.5 Hz, 1H), 4.58 (q, J = 2.7, 1H), 3.46 (dd, J_1 = 2.7 Hz, J_2 = 9.8 Hz, 1H), 3.21 (dd, J_1 = 2.7 Hz, J_2 = 9.9 Hz, 1H); ^{13}C NMR (400 MHz, DMSO-d_6) δ 170.98, 148.65, 148.52, 146.15, 146.01, 141.99, 141.85, 141.41, 139.54, 139.40, 118.09, 118.06, 111.20, 111.18, 110.89, 100.85, 104.15, 104.12, 104.01, 103.97, 88.62, 88.47, 51.83, 24.82; ^{19}F NMR (400 MHz, DMSO-d_6) δ -134.73, -134.79, -148.27, -148.33; ESI-MS: Calcd. for $C_{11}H_4F_2N_3O_2S$ $[M-H]^+$ 280.0; found 280.2.

1.3 Procedure for fluorescence measurement

1.3.1 Procedure for sensitivity and selectivity test

Appropriate amount of the probe (4F-2CN) was dissolved in DMSO to prepare 1 mM stock

solution. The probe was then diluted in PB buffer (10 mM, pH 7.4) to afford a final concentration of 10 μM. The amino acids were prepared as 100 mM or 10 mM stock solutions in PB buffer (10 mM, pH 7.4). For sensitivity experiments, different concentrations of Cys/Hcy/GSH were added to separate portions of the probe solution and mixed thoroughly. For selectivity experiments, appropriate amount of Cys/Hcy/GSH and other biological analytes were added to separate portions of the probe solution. The reaction mixture was shaken uniformly at 37°C for 2h before emission spectra measurement. The fluorescence spectra were collected using a FluoroMax-4 fluorescence photometer with a 10 mm quartz cuvette.

1.3.2 Procedure for kinetic test

The probe, 4F-2CN, was dissolved in DMSO to prepare 10 mM stock solution. Cys/Hcy/GSH were dissolved in PB buffer to prepare 100 mM stock solution. The probe and the corresponding thiols were then diluted in PB buffer (10 mM, pH 7.4) to afford 3 mL of solution with final concentration of 10 μM 4F-2CN and 100 μM Cys/Hcy/GSH individually. The fluorescence intensity of the reaction mixture was measured at different time points.

1.3.3 Detection Limit

Detection limit or limit of detection (LOD) was estimated from the standard deviation of the blank and the corresponding linear regression equation. Specifically we used the following equation in this study [1]:

$$\mathrm{LOD} = 3\sigma/m,$$

$$\sigma = \sqrt{\frac{\sum(\bar{x}-xi)^2}{n-1}}$$

$\bar{x}$ is the mean of the blank measures; xi is the values of blank measures; n is the number of tested blank measure (n = 11). m is the slope of the linear regression equation derived from

fluorescence intensity and concentration.

1.3.4 Determination of quantum yield

The quantum yields of the fluorophores were calculated by comparing the integrated area of the emission spectrum of the sample with that of a reference solution. Specifically, the quantum yield was determined by the following equation [2]:

$$\emptyset_{sample} = \left(\frac{I_{reference}}{I_{sample}}\right)\left(\frac{A_{sample}}{A_{reference}}\right)\emptyset_{reference}$$

Where Φ is quantum yield; I is the absorption intensity at the excitation wavelength; A is the integrated fluorescent intensity. In this study, Coumarin 6 in absolute ethanol was used as the standard, which has a quantum yield of 0.78.

2. Supplementary Figures

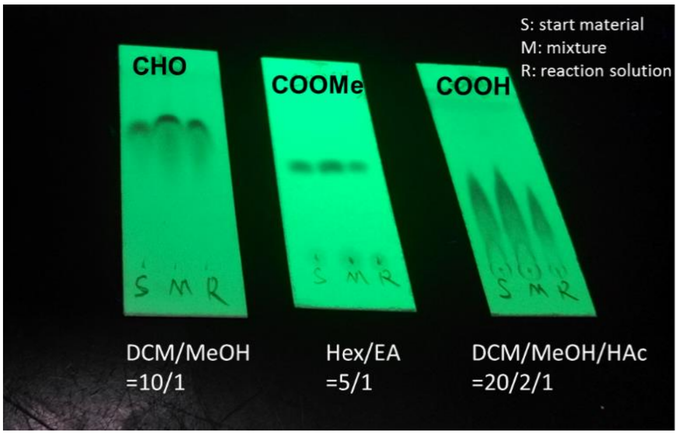

Fig. S1 TLC experiments of 4F-2CN analogues with Cys. 4F-2CN analogues and Cys were mixed in DMF/PBS = 1:1, incubated for 2h. Results indicated that no reaction has occurred between the analogues and Cys.

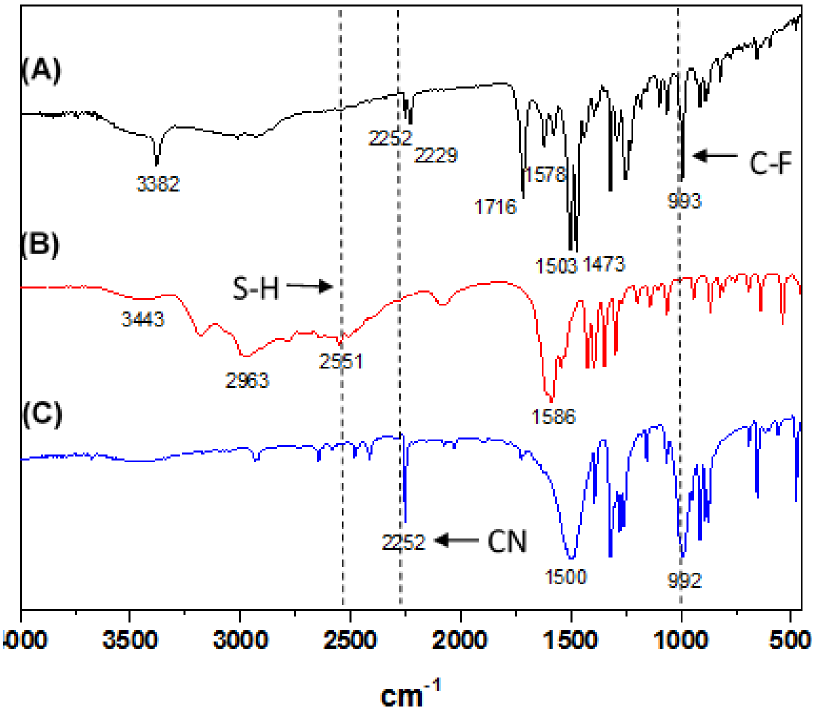

Fig. S2 FT-IR spectra of 4F-2CN-Cys (A), Cys (B) and 4F-2CN (C). 4F-2CN-Cys shows several characteristic IR absorption peaks, e.g. the peak at 2252 cm−1 (CN) and 992 cm−1 (C-F stretching). In addition, the peak at 2551 cm−1, which belongs to S-H in cysteine, has disappeared in 4F-2CN-Cys, indicating that nucleophilic substitution has occurred.

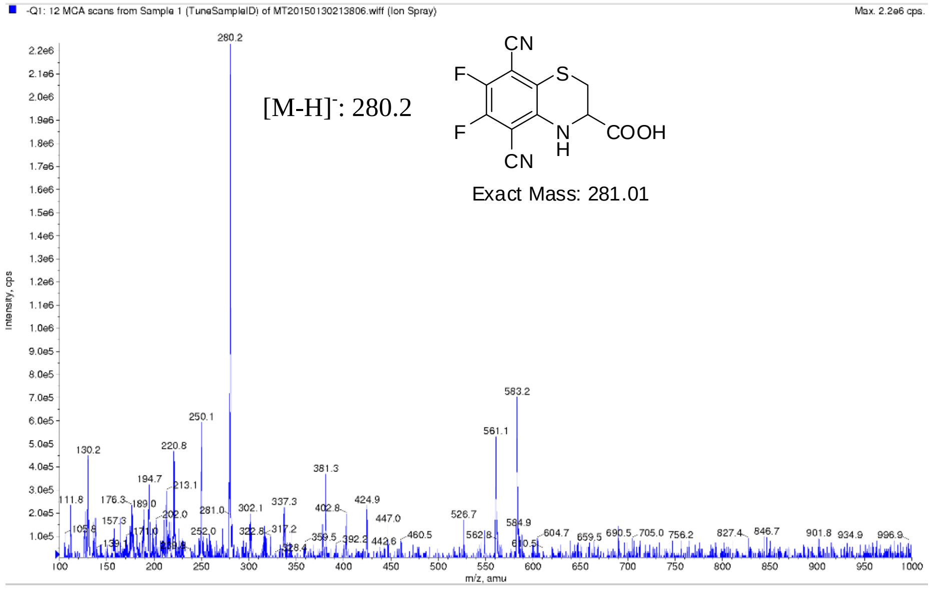

Fig. S3 MS spectrum of **4F-2CN-Cys**.

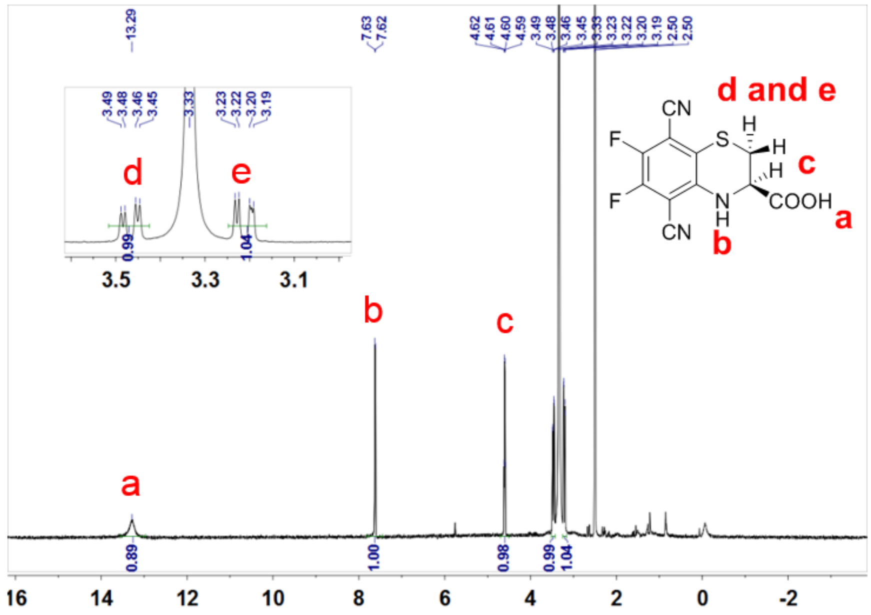

Fig. S4 ^{1}H NMR spectrum of **4F-2CN-Cys**. The chemical shift at 13.29 ppm (a) represents the proton of the carboxylic acid group. The chemical shift at 7.62 ppm (b) belongs to the proton of the amine group. The chemical shift at 4.60 ppm (c) represents the proton next to an amine group. The chemical shift at 3.47 and 3.21 ppm belong to proton d and proton e respectively. The two protons are split into dd pattern due to the adjacent chiral proton.

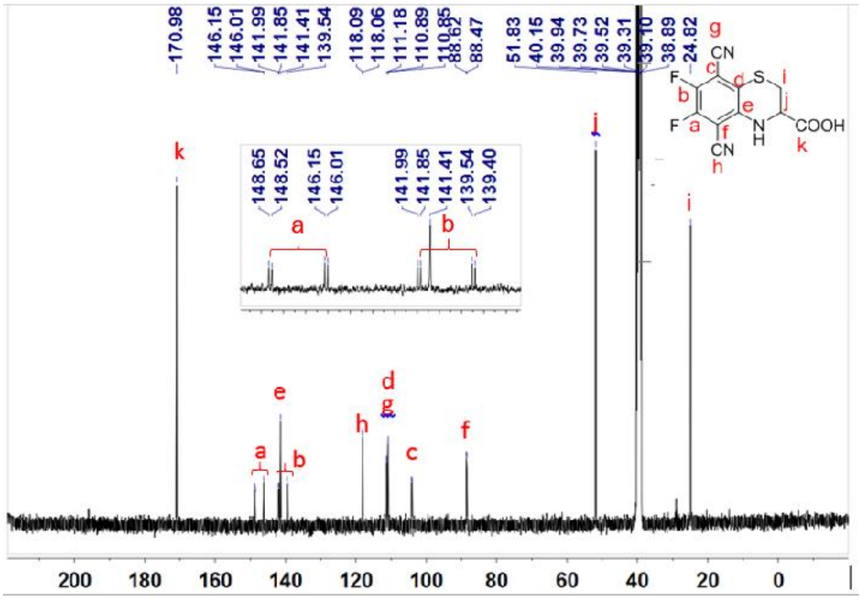

Fig. S5 ^{13}C NMR spectrum of **4F-2CN-Cys**. The four peaks at chemical shift from 148.65 to 146.01 ppm belong to one carbon. The splitting of the carbon signal is due to the coupling of the carbon and the two adjacent fluorine atoms. Similarly, the four peaks at chemical shift from 141.99 to 139.40 ppm belong to one carbon.

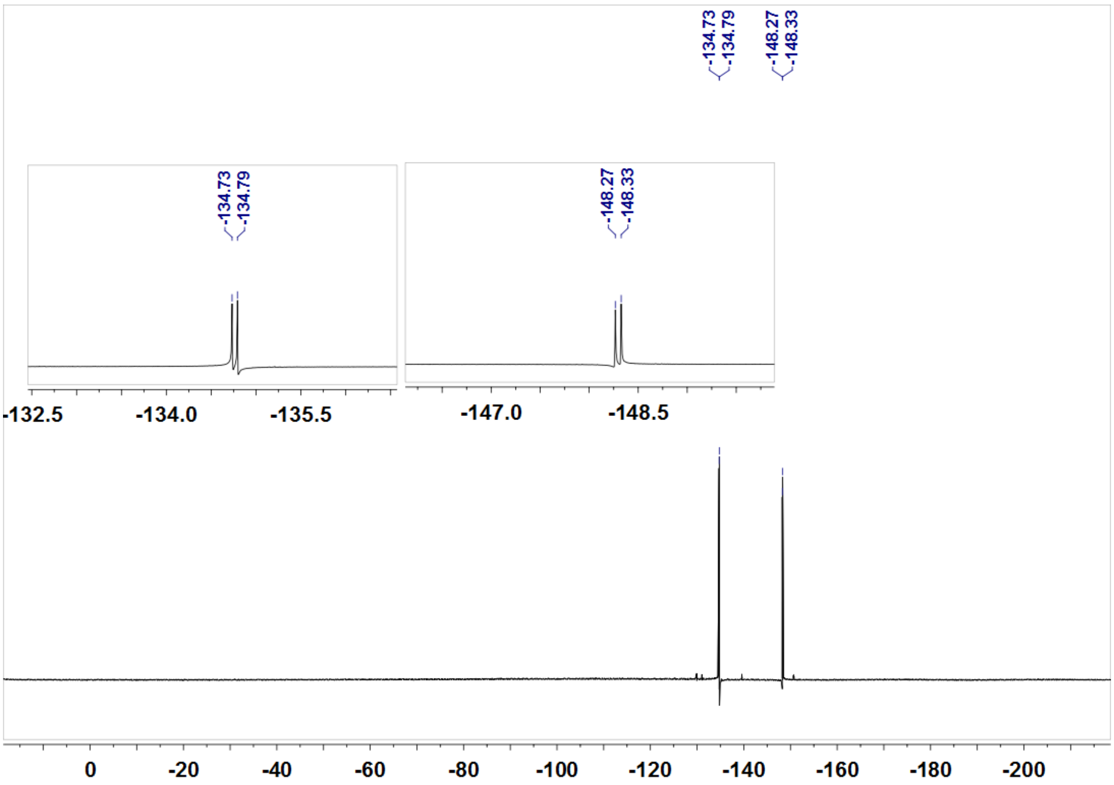

Fig. S6 ^{19}F NMR spectrum of **4F-2CN-Cys**. The spectrum shows two doublet peaks at 134 and 148 ppm. Because the molecule is unsymmetrical, the two fluorine atoms of 4F-2CN show different chemical shift. Each fluorine signal is split into two peaks by the adjacent fluorine atom.

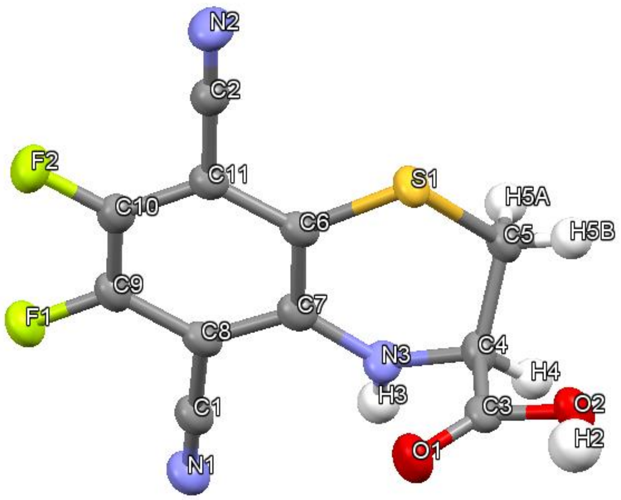

Fig. S7 X-ray structure of **4F-2CN-Cys**. Color codes: oxygen, red; nitrogen, blue; carbon, light gray; fluorine, green; sulfur, yellow; hydrogen, white.

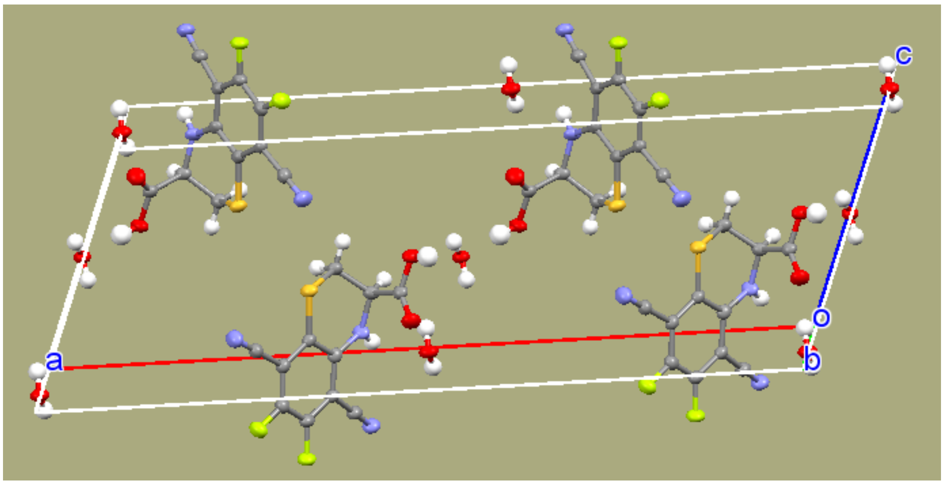

Fig. S8 Unit cell packing diagram of **4F-2CN-Cys**. Color codes: oxygen, red; nitrogen, blue; carbon, light gray; fluorine, green; sulfur, yellow; hydrogen, white.

Table S1. Selected crystallographic data for **4F-2CN-Cys**.

Compound	**4F-2CN-Cys**
Chemical formula	$C_{11}H_5F_2N_3O_2S$, H_2O
Formula Mass	299.26

Crystal system	monoclinic
a, Å	26.261(3)
b, Å	5.1620(4)
c, Å	9.7804(10)
α, deg	90.0
β, deg	111.188(12)
γ, deg	90.0
V, $Å^3$	1236.2(2)
T/K	173(2)
space group	C121
Z	4
ρ_{calcd}, g/cm^3	1.608
wavelength, Å	1.54178 (Cu Kα)
abs coeff (μ), mm^{-1}	2.715
Crystal size	0.41*0.06*0.01 mm
Final R indices [**I** >2σ(**I**)]	R1=0.0293, wR2=0.0308
R indices (all data)	R1=0.0857, wR2=0.0848
CCDC number	CCDC 1402255

Table S2. Selected bond lengths (Å) for **4F-2CN-Cys**.

S(1)-C(5)	1.805(3)	S(1)-C(6)	1.754(2)
F(1)-C(9)	1.335(3)	F(2)-C(10)	1.342(3)
O(1)-C(3)	1.203(4)	O(2)-H(2)	0.8400
O(2)-C(3)	1.313(3)	N(1)-C(1)	1.144(4)

N(2)-C(2)	1.149(4)	N(3)-H(3)	0.8800
N(3)-C(4)	1.441(3)	N(3)-C(7)	1.371(3)
C(1)-C(8)	1.432(4)	C(2)-C(11)	1.431(4)
C(3)-C(4)	1.524(4)	C(4)-H(4)	1.0000
C(4)-C(5)	1.520(3)	C(5)-H(5A)	0.9900
C(5)-H(5B)	0.9900	C(6)-C(7)	1.421(4)
C(6)-C(11)	1.397(4)	C(7)-C(8)	1.416(3)
C(8)-C(9)	1.393(4)	C(9)-C(10)	1.367(4)
C(10)-C(11)	1.387(4)		

Table S3. Selected bond angles (°) for **4F-2CN-Cys**.

C(6)-S(1)-C(5)	100.08(14)	C(3)-O(2)-H(2)	109.5
C(4)-N(3)-H(3)	118.5	C(7)-N(3)-H(3)	118.5
C(7)-N(3)-C(4)	123.0(2)	N(1)-C(1)-C(8)	177.9(3)
N(2)-C(2)-C(11)	179.2(3)	O(1)-C(3)-O(2)	124.1(2)
O(1)-C(3)-C(4)	124.7(2)	O(2)-C(3)-C(4)	111.1(2)
N(3)-C(4)-C(3)	112.2(2)	N(3)-C(4)-H(4)	107.7
N(3)-C(4)-C(5)	111.6(2)	C(3)-C(4)-H(4)	107.7
C(5)-C(4)-C(3)	109.8(2)	C(5)-C(4)-H(4)	107.7
S(1)-C(5)-H(5A)	109.4	S(1)-C(5)-H(5B)	109.4
C(4)-C(5)-S(1)	111.28(18)	C(4)-C(5)-H(5A)	109.4
C(4)-C(5)-H(5B)	109.4	H(5A)-C(5)-H(5B)	108.0
C(7)-C(6)-S(1)	122.7(2)	C(11)-C(6)-S(1)	118.1(2)
N(3)-C(7)-C(6)	123.0(2)	N(3)-C(7)-C(8)	119.7(2)
C(8)-C(7)-C(6)	117.3(2)	C(7)-C(8)-C(1)	121.0(2)

C(9)-C(8)-C(1)	117.5(2)	C(9)-C(8)-C(7)	121.4(2)
F(1)-C(9)-C(8)	119.2(2)	F(1)-C(9)-C(10)	120.2(2)
C(10)-C(9)-C(8)	120.6(2)	F(2)-C(10)-C(9)	121.0(2)
F(2)-C(10)-C(11)	119.9(2)	C(9)-C(10)-(C11)	119.2(2)
C(6)-C(11)-C(2)	119.7(2)	C(10)-C(11)-C(2)	118.1(2)
C(10)-C(11)-C(6)	122.1(2)		

Table S4. Quantum yield of 4F-2CN-Cys, 4F-2CN and coumarin 6.

Compound	λ_{ex}	λ_{em}	ε	Φ
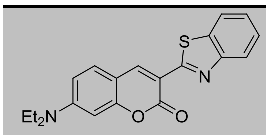 Coumarin 6	460 nm	500 nm	5.34×10^{4}	0.78
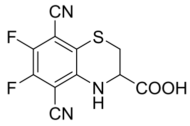 4F-2CN-Cys	420 nm	500 nm	1.34×10^{4}	0.35
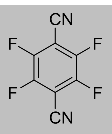 4F-2CN	312 nm	350 nm	3.29×10^{3}	ND

ND = "not determined"

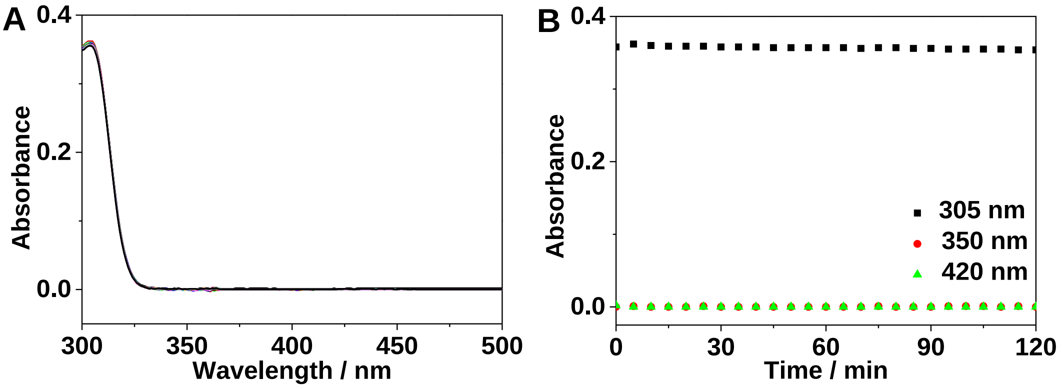

Fig. S9 (A) Absorption spectrum of 4F-2CN (50 μM) in PB buffer (10 mM, pH 7.4). (B) Time-dependent absorbance changes of 4F-2CN (50 μM) in PB buffer monitored at three different wavelengths (305 nm, 350 nm and 420 nm).

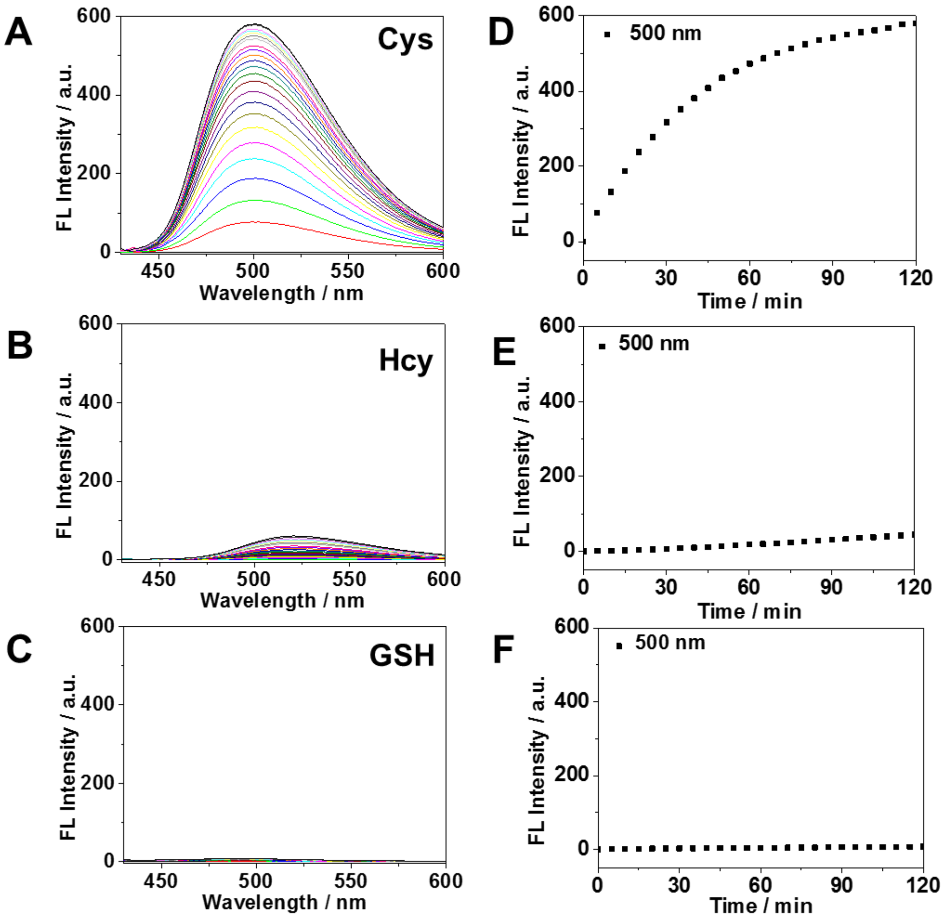

Fig. S10 Time-dependent fluorescence spectra of 4F-2CN (10 μM) with 100 μM of Cys (A), Hcy (B) and GSH (C) excited at 420 nm respectively (PB buffer, pH 7.4, 10 mM). Corresponding fluorescence intensity changes of 4F-2CN with Cys (D), Hcy (E) and GSH (F) excited at 420 nm respectively.

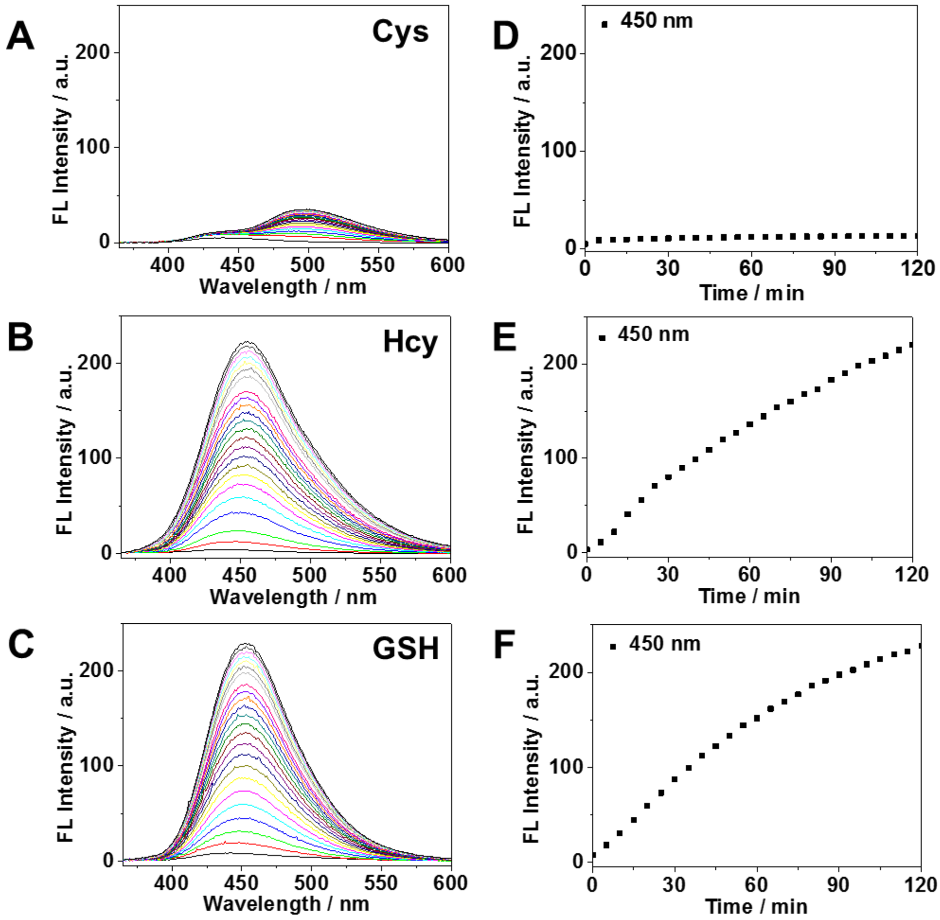

Fig. S11 Time-dependent fluorescence spectra of 4F-2CN (10 μM) with 100 μM of Cys (A), Hcy (B) and GSH (C) excited at 350 nm respectively (PB buffer, pH 7.4, 10 mM)). Corresponding fluorescence intensity changes of 4F-2CN with Cys (D), Hcy (E) and GSH (F) excited at 350 nm respectively.

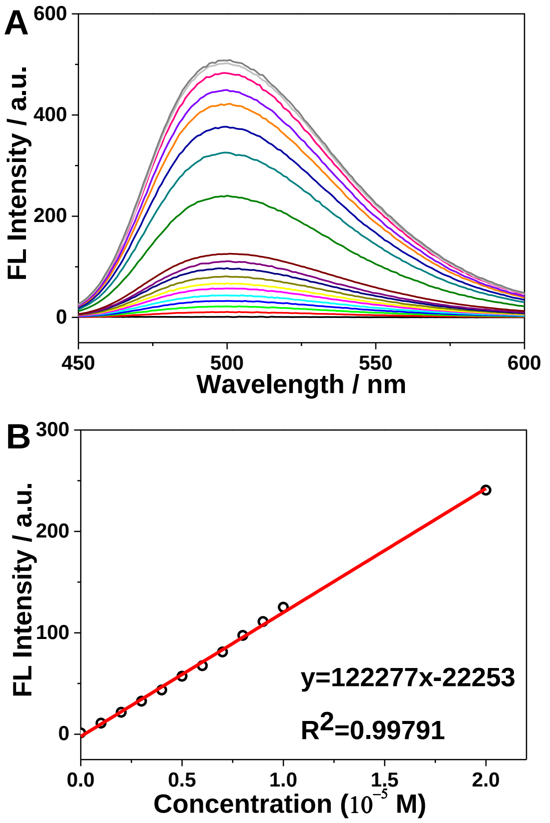

Fig. S12 Fluorescence response of 4F-2CN with addition of various concentrations of Cys. The fluorescence intensity was measured using Ex/Em = 420 / 500 nm.

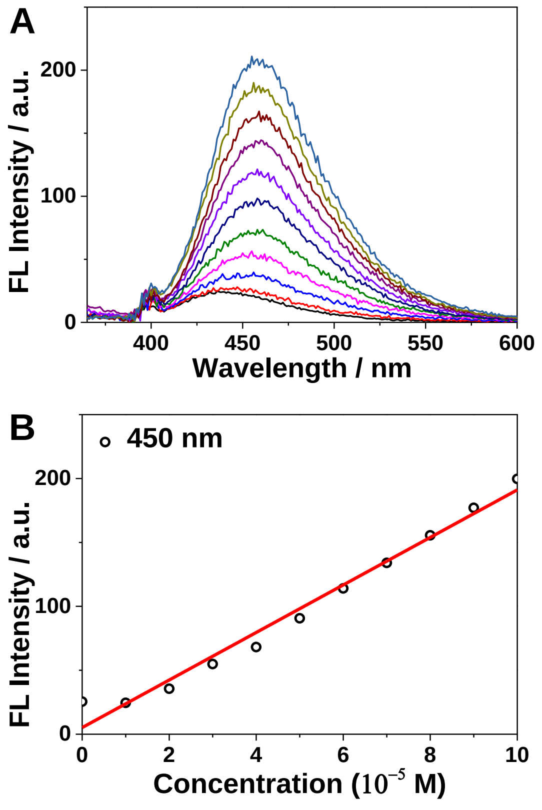

Fig. S13 Fluorescence response of 4F-2CN with addition of various concentrations of HCy. The fluorescence intensity was measured using Ex/Em = 350 / 450 nm.

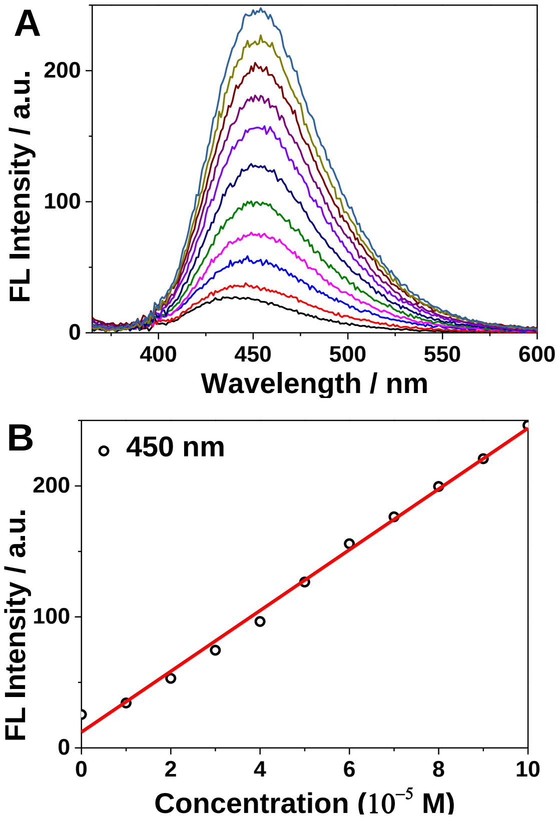

Fig. S14 Fluorescence response of 4F-2CN with addition of various concentrations of GSH. The fluorescence intensity was measured using Ex/Em = 350 / 450 nm.

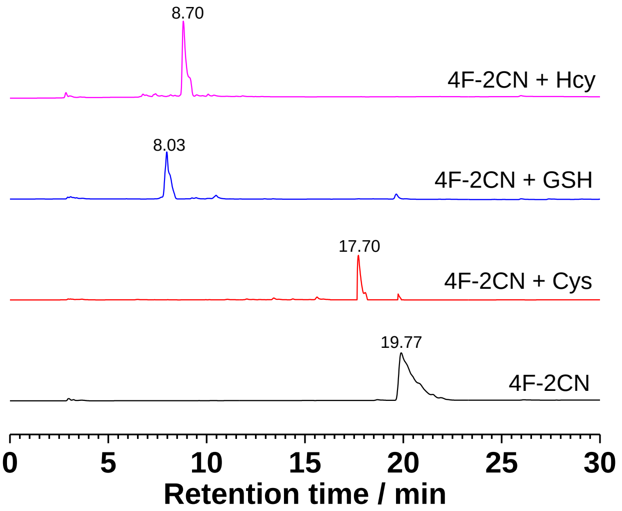

Fig. S15 HPLC profiles of the reactions of 4F-2CN with Cys, Hcy and GSH after 2h of incubation. HPLC conditions: 1 mL/min flow rate, 10% B to 100% B over 30 min, detected at 254 nm. Solvent A is water with 0.1% trifluoroacetic acid, and solvent B is acetonitrile with 0.1% trifluoroacetic acid.

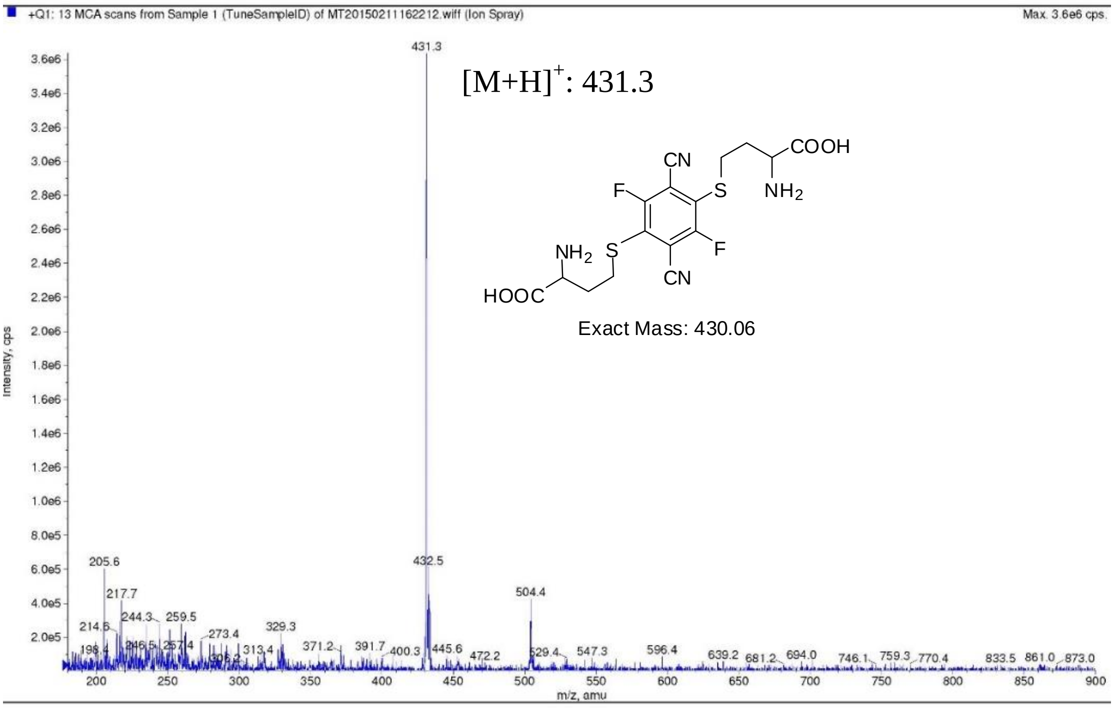

Fig. S16 Mass spectrum of the reaction product of 4F-2CN and Hcy.

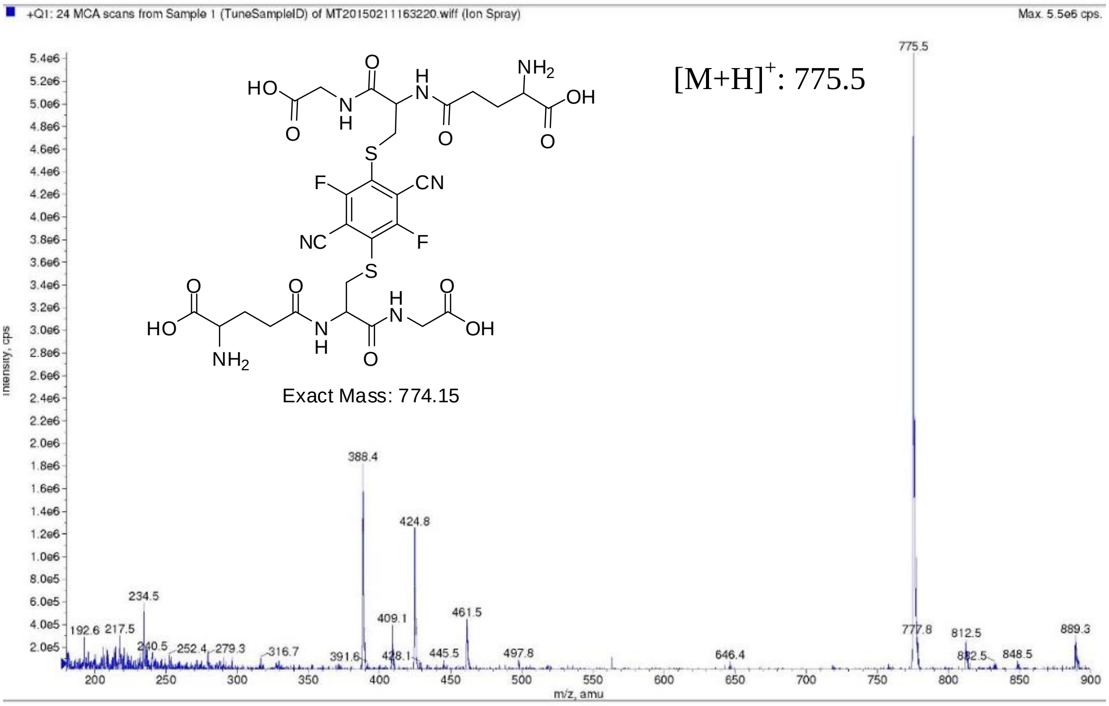

Fig. S17 Mass spectrum of the reaction product of 4F-2CN and GSH.

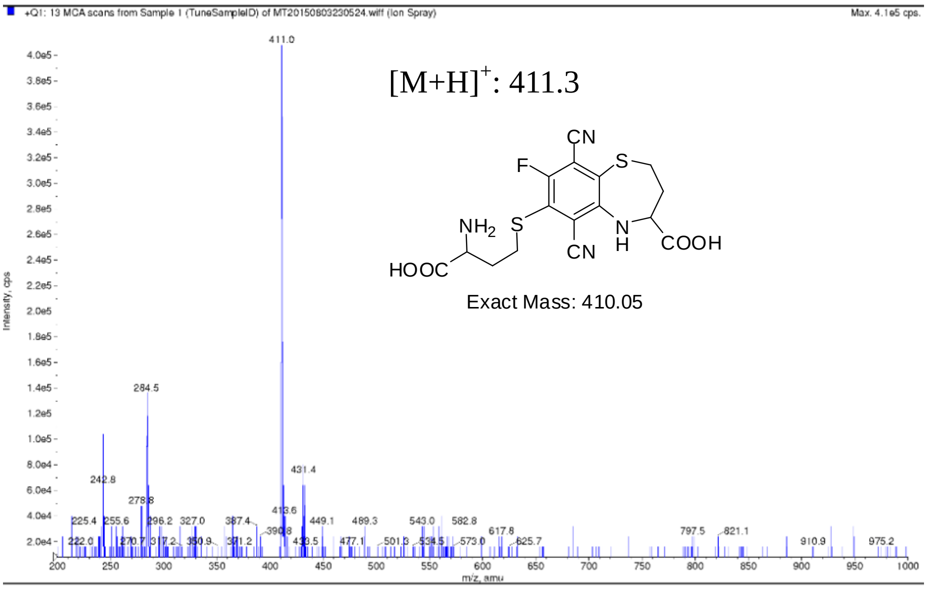

Fig. S18 Mass spectrum of the reaction product of 4F-2CN, Hcy and CTAB.

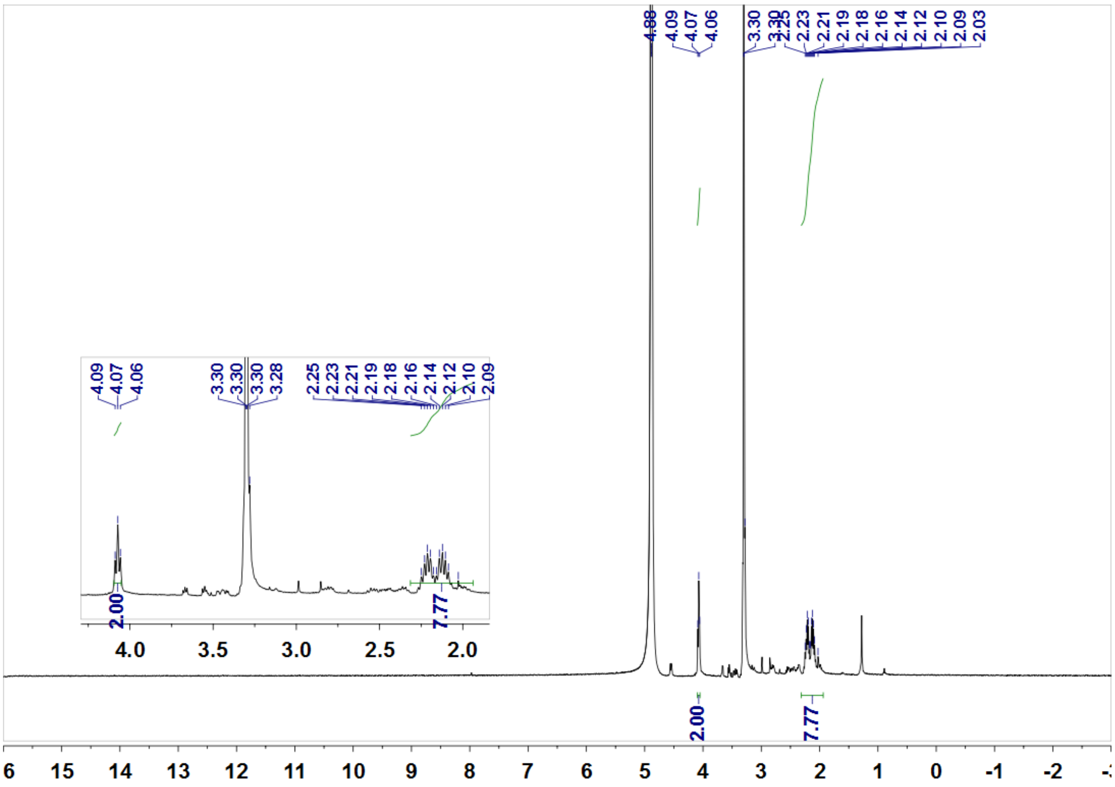

Fig. S19 ^{1}H NMR spectrum of **4F-2CN-Hcy**.

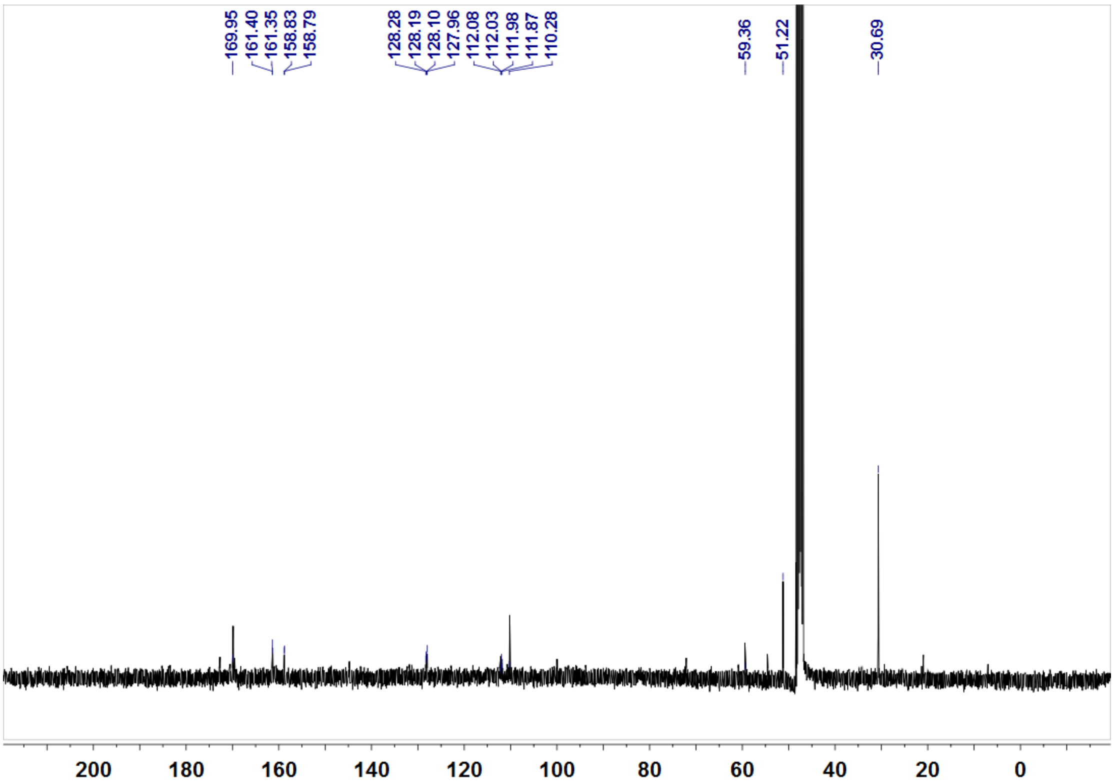

Fig. S20 ^{13}C NMR spectrum of **4F-2CN-Hcy**.

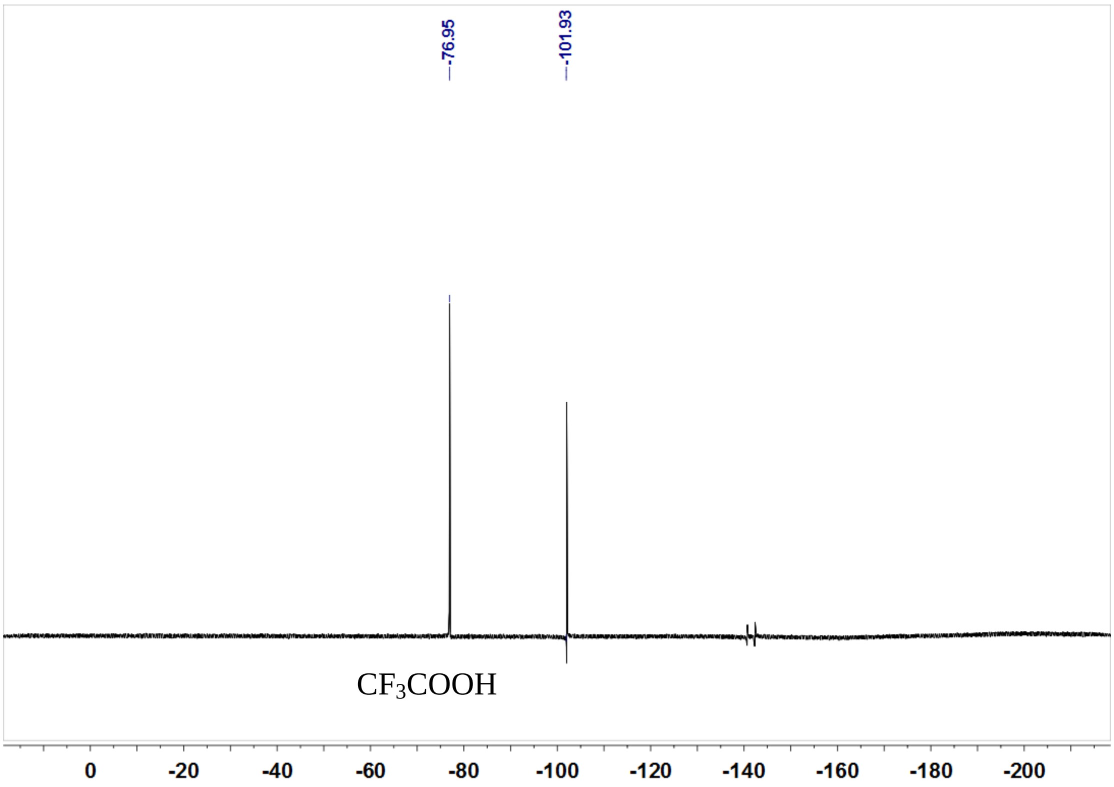

Fig. S21 ^{19}F NMR spectrum of **4F-2CN-Hcy**.

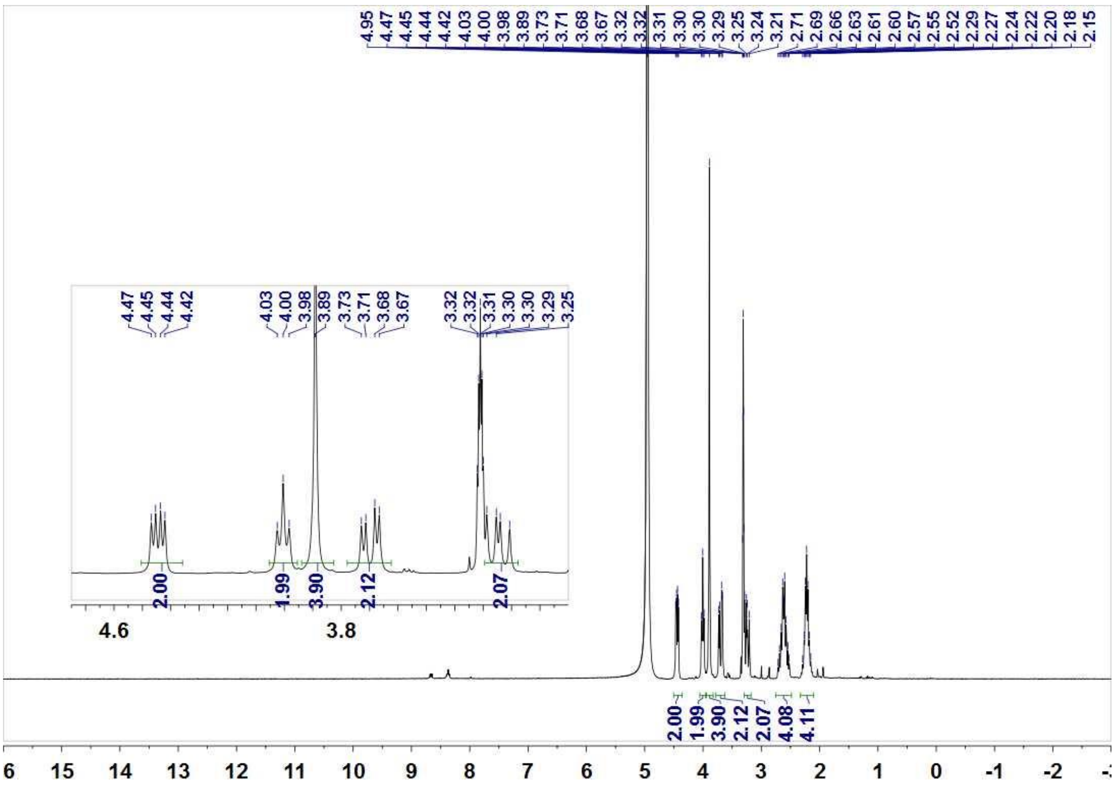

Fig. S22 ^{1}H NMR spectrum of **4F-2CN-GSH.**

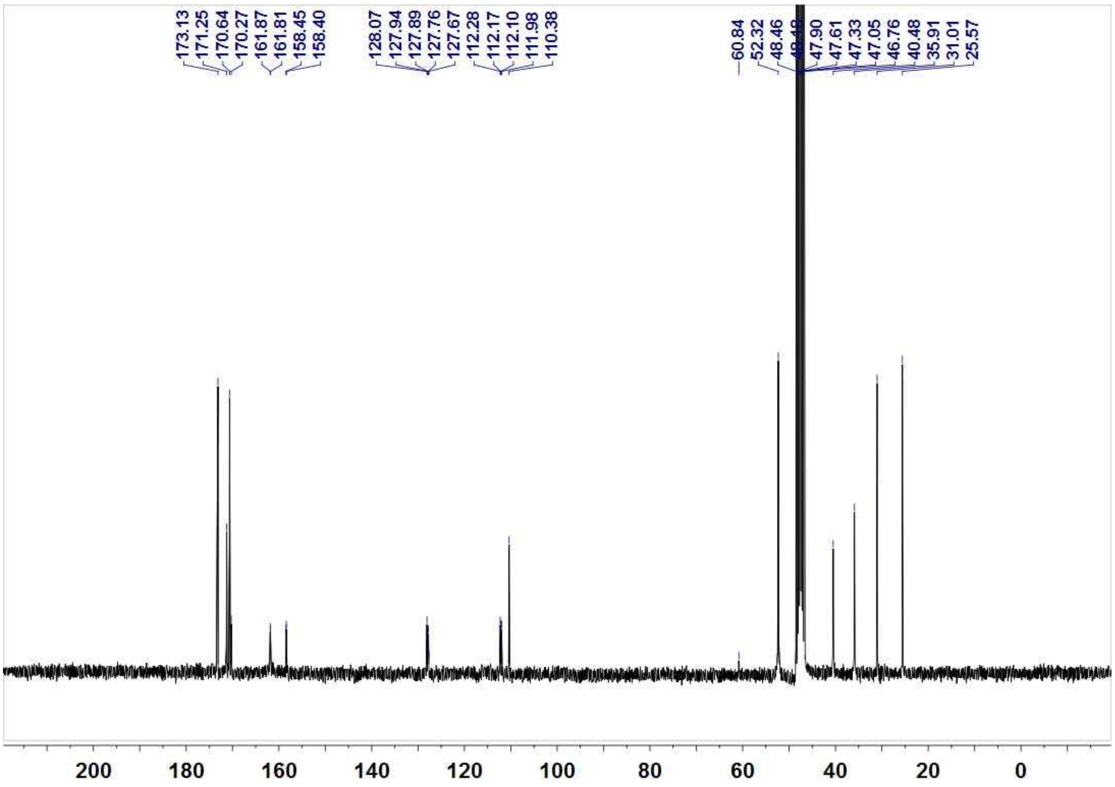

Fig. S23 ^{13}C NMR spectrum of **4F-2CN-GSH**.

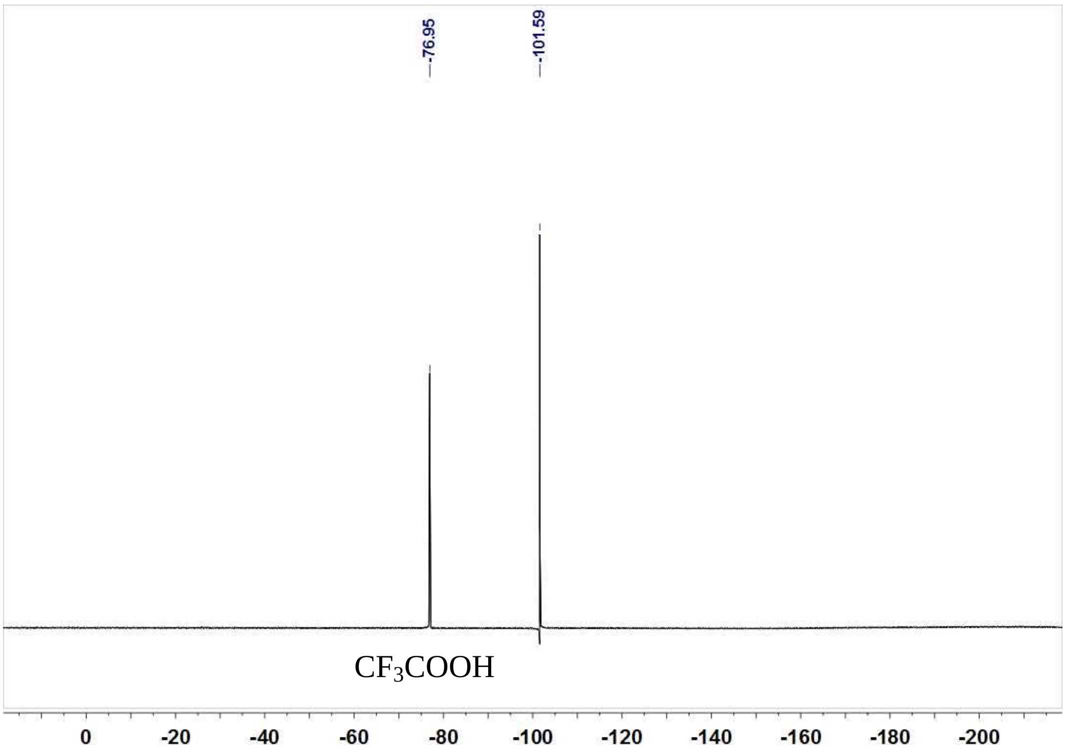

Fig. S24 ^{19}F NMR spectrum of **4F-2CN-GSH**.

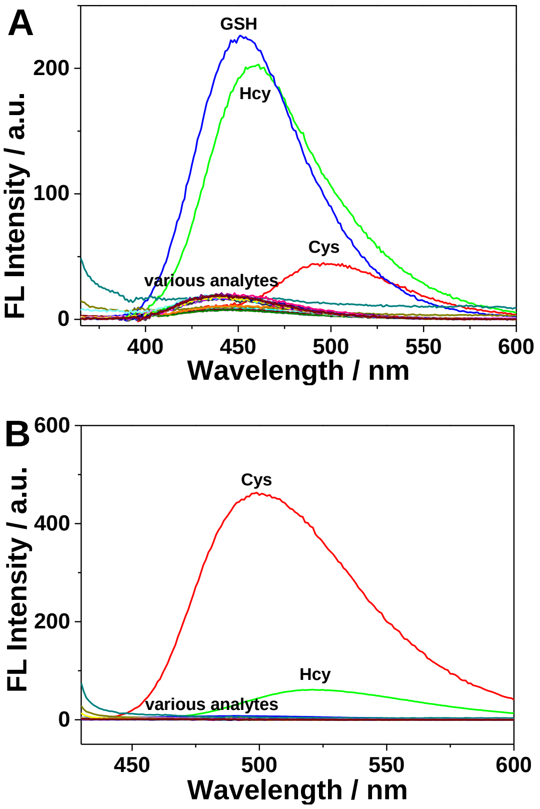

Fig. S25 Selectivity experiments with Cys, Hcy, GSH and other different biological analytes. (A) Fluorescence intensity was measured at an excitation wavelength of 350 nm; (B) Fluorescence intensity was measured at an excitation wavelength of 420 nm. Various analytes include: K^+, Na^+, Mg^{2+}, Zn^{2+}, NO_2^-, N_3^-; $S_2O_3^{2-}$, S^{2-}, •OH, •OtBu, H_2O_2, TBHP, Ala, Ile, Leu, Val, Phe, Try, Tyr, Asn, Gln, Met, Ser, Thr, Asp, Glu, Arg, His, Lys, Gly, Pro.

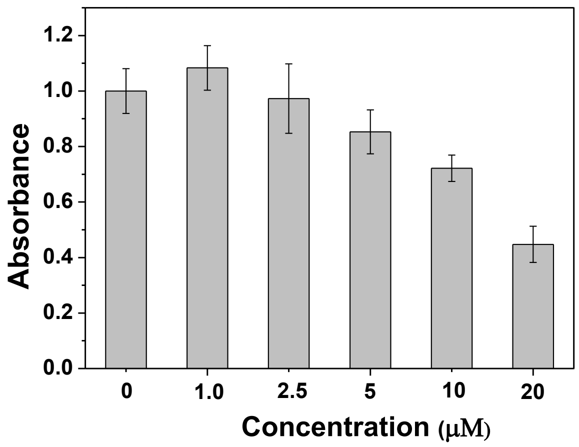

Fig. S26 MTT assay with different concentrations of 4F-2CN in A549 cells after 24 h incubation at 37 ^{o}C.

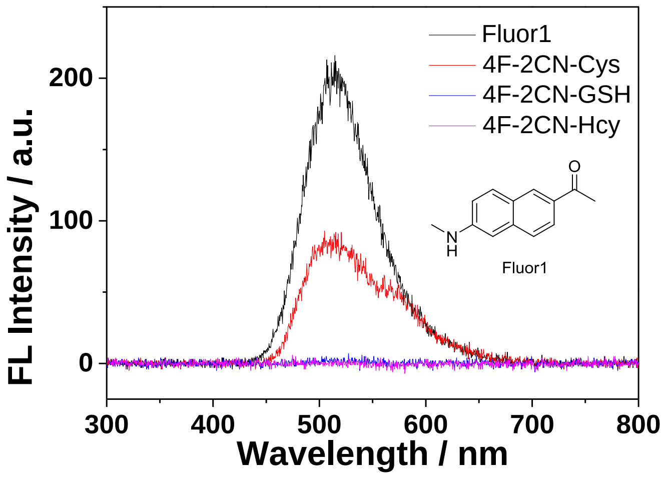

Fig. S27 Two-photon excited fluorescence emission spectra of **Fluor1, 4F-2CN-Cys, 4F-2CN-GSH** and **4F-2CN-Hcy** in PB buffer (Ex: 860 nm). Fluor1 is a two-photon fluorophore commonly used for cell imaging studies.

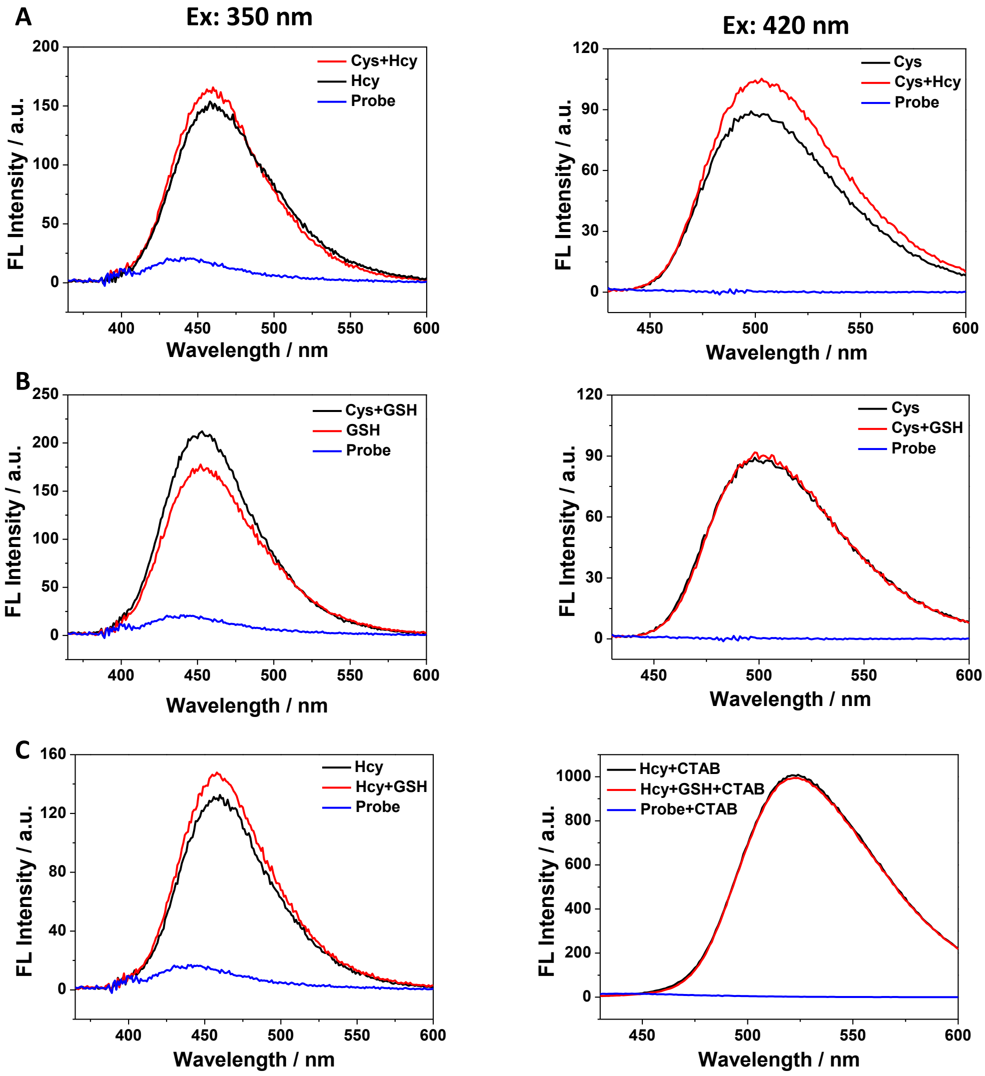

Fig. 28 Fluorescence response of 4F-2CN (20 μM) with thiol mixtures. (A) Fluorescence spectra of 4F-2CN in the presence of Cys/Hcy (10 μM/100 μM) excited at 350 nm and 420 nm respectively; (B) Fluorescence spectra of 4F-2CN in the presence of Cys/GSH (10 μM/100 μM) excited at 350 nm and 420 nm respectively; (C) Fluorescence spectra of 4F-2CN in the presence of GSH/Hcy (10 μM/100 μM) excited at 350 nm and 420 nm respectively.

3. References

[1] H. Zhang, R. Liu, Y. Tan, W.-H. Xie, H. Lei, H.-Y. Cheung and H. Sun, *ACS Appl. Mater. Interfaces.* **2015**, *7*, 5438-5443.

[2] T. Peng, N. K. Wong, X. Chen, Y. K. Chan. D. H. Ho, Z. Sun, J. J. Hu, J. Shen, H. El-Nezami,

D. Yang, *J. Am. Chem. Soc.* **2014**, *136*, 11728-11734.
